# Comparisons of intellectual capacities between mild and classic adult-onset phenotypes of myotonic dystrophy type 1 (DM1)

**DOI:** 10.1186/s13023-014-0186-5

**Published:** 2014-11-26

**Authors:** Stéphane Jean, Louis Richer, Luc Laberge, Jean Mathieu

**Affiliations:** Clinique des maladies neuromusculaires, Centre de réadaptation en déficience physique Le Parcours, Centre de santé et de services sociaux de Jonquière, 2230, rue de l’Hôpital, C.P. 1200, Jonquière, Québec G7X 7X2 Canada; Département des sciences de la santé, Université du Québec à Chicoutimi, 555, boul. de l’Université, Chicoutimi, Québec G7H 2B1 Canada; ÉCOBES - Recherche et transfert, Cégep de Jonquière, Québec, Canada; Faculté de médecine et des sciences de la santé, Université de Sherbrooke, Québec, Canada

**Keywords:** Myotonic dystrophy, Phenotype, Central nervous system, Neuropsychology, Intellectual disability, Dystrophie myotonique, Phénotype, Système nerveux central, Neuropsychologie, Déficience intellectuelle

## Abstract

**Background:**

Myotonic dystrophy type 1 (DM1) is an autosomal dominant genetic multisystem disorder and the commonest adult-onset form of muscular dystrophy. DM1 results from the expansion of an unstable trinucleotide cytosine-thymine-guanine (CTG) repeat mutation. CTG repeats in DM1 patients can range from 50 to several thousands, with a tendency toward increased repeats with successive generations (anticipation). Associated findings can include involvements in almost every systems, including the brain, and cognitive abnormalities occur in the large majority of patients. The objectives are to describe and compare the intellectual abilities of a large sample of DM1 patients with mild and classic adult-onset phenotypes, to estimate the validity of the Wechsler Adult Intelligence Scale-Revised (WAIS-R) in DM1 patients with muscular weakness, and to appraise the relationship of intelligence quotient (IQ) to CTG repeat length, age at onset of symptoms, and disease duration.

**Methods:**

A seven-subtest WAIS-R was administered to 37 mild and 151 classic adult-onset DM1 patients to measure their Full-Scale (FSIQ), Verbal (VIQ) and Performance IQ (PIQ). To control for potential bias due to muscular weakness, Standard Progressive Matrices (SPM), a motor-independent test of intelligence, were also completed.

**Results:**

Total mean FSIQ was 82.6 corresponding to low average IQ, and 82% were below an average intelligence. Mild DM1 patients had a higher mean FSIQ (*U*=88.7 vs 81.1, *p*<0.001), VIQ (*U*=87.8 vs 82.3, *p*=0.001), and PIQ (*U*=94.8 vs 83.6, *p*<0.001) than classic adult-onset DM1 patients. In both mild and classic adult-onset patients, all subtests mean scaled scores were below the normative sample mean. FSIQ also strongly correlate with SPM (*r*_*s*_=0.67, *p*<0.001), indicating that low intelligence scores are not a consequence of motor impairment. FSIQ scores decreased with both the increase of (CTG)n (*r*_*s*_=−0.41, *p*<0.001) and disease duration (*r*_*s*_=−0.26, *p*=0.003).

**Conclusions:**

Results show that intellectual impairment is an extremely common and important feature in DM1, not only among the classic adult-onset patients but also among the least severe forms of DM1, with low IQ scores compared to general reference population. Health care providers involved in the follow-up of these patients should be aware of their intellectual capacities and should adapt their interventions accordingly.

## French abstract

**Contexte:**

La dystrophie myotonique de type 1 (DM1) est une maladie génétique à transmission autosomique dominante et la dystrophie musculaire la plus fréquemment diagnostiquée chez l’adulte. Elle résulte d’une répétition instable d’un trinucléotide cytosine-thymine-guanine (CTG). Le nombre de CTG peut varier de 50 à plusieurs milliers. Elle affecte pratiquement tous les systèmes, incluant le cerveau, et des déficits cognitifs surviennent chez une majorité de patients. Les objectifs sont de décrire et comparer les capacités intellectuelles d’un large échantillon de patients atteints de la forme légère et adulte-classique de DM1, d’estimer la validité de l’Échelle d’intelligence de Wechsler-Révisée (WAIS-R) chez des patients présentant une faiblesse musculaire, et d’évaluer la relation entre le quotient intellectuel (QI) et le nombre de CTG, l’âge d’apparition des symptômes et la durée de la maladie.

**Méthodes:**

Un WAIS-R à sept sous-tests fut administré à 37 patients légers et 151 adultes-classiques pour estimer leur QI global, Verbal et Performance. Afin de contrôler des biais potentiels liés à la faiblesse musculaire, les Matrices de Raven ont également été complétées.

**Résultats:**

Au total, le QI global moyen était de 82.6, correspondant à un QI situé dans la moyenne faible, et 82% a obtenu un QI global inférieur à une intelligence moyenne. Les patients légers ont obtenu des résultats moyens supérieurs aux patients adultes-classiques au QI global (*U* = 88.7 vs 81.1, *p* < 0.001), Verbal (*U* = 87.8 vs 82.3, *p* = 0.001) et Performance (*U* = 94.8 vs 83.6, *p* < 0.001). Pour les deux groupes, les scores moyens aux sous-tests étaient tous situés sous la moyenne normative. Le QI global corrèle fortement avec les Matrices (*r*_*s*_ = 0.67, *p* < 0.001), ce qui montre que l’atteinte musculaire n’influence pas la mesure de l’intelligence. Le QI global diminue avec l’augmentation des CTG (*r*_*s*_ = -0.41, *p* < 0.001) et la durée de la maladie (*r*_*s*_ = -0.26, *p* = 0.003).

**Conclusions:**

L’altération du fonctionnement intellectuel marquée par de faibles QI est une caractéristique importante et extrêmement fréquente dans la DM1, non seulement dans la forme adulte-classique, mais également dans la forme moins sévère. Les professionnels de la santé impliqués dans le suivi de ces patients devraient tenir compte de leurs capacités intellectuelles et adapter leurs interventions en conséquence.

## Background

Myotonic dystrophy type 1 (DM1) is an autosomal dominant genetic disorder with high but incomplete penetrance and the commonest adult-onset form of muscular dystrophy. As a muscle disease, DM1 is characterized by an inability to relax voluntary muscle contractions (myotonia) and by progressive distal to proximal muscle weakness. Associated findings can include involvements in almost every systems such as the cardiac, respiratory, gastrointestinal, endocrine, ocular, and central nervous system (CNS) [1]. DM1 results from the expansion of an unstable trinucleotide cytosine-thymine-guanine (CTG) repeat mutation located in the 3′ untranslated region of a gene (19q13.3) encoding a putative protein kinase (*DMPK*) [2]. When transcribed into CUG-containing RNA, mutant transcripts aggregate as nuclear foci that sequester RNA-binding proteins, including members of the muscleblind (MBNL) family, resulting in a spliceopathy of downstream effector genes [3]. CTG repeats in DM1 patients can range from 50 to several thousands, with a tendency toward increased repeats with successive generations (anticipation). The length of the CTG repeats is partly correlated to the severity of the disease and the age at onset of symptoms [4]. Considering various European populations, the worldwide prevalence of DM1 could be estimated at 5–20 per 100 000 [1]. In 2010, the highest prevalence reaches 158 per 100 000 in the Saguenay-Lac-Saint-Jean (SLSJ) region located in the eastern part of the province of Quebec (Canada) [5].

Involvement of the CNS occurs in the large majority of patients with DM1 [1,6,7], particularly when symptoms appear early in life. This can range from a condition of intellectual disability (a characteristic often associated with congenital DM1 in which symptoms are manifest from birth), to behavioural changes (e.g. reduced initiative, inactivity, apathetic temperament) [1]. Higher cognitive function disabilities are variably impaired [7–13] but a trend toward reduced frontal lobe performances has often been reported, along with many significant brain changes (e.g. brain atrophy, cell loss, ventricular enlargement, diffuse white matter lesions as well as significant cerebral blood flow reduction in frontotemporal lobe regions) [1,14]. Excessive daytime sleepiness is a prominent feature of DM1 and is most often considered as independent from respiratory dysfunction or nocturnal sleep disruption [15,16]. Moreover, psychopathological disturbances such as avoidant, dependent and paranoid personality traits are frequent in adult form of DM1 [17,18].

Neuropsychological studies have shown evidences for a generally lower intelligence level in the DM1 population as compared to normal control subjects [13,14,19–21], with no clear evidence of a progressive intellectual decline over time [7,8,20,22,23]. The highest prevalence of intellectual impairment has been reported in studies that have included both patients with adult and congenital onset of DM1, even if it is commonly accepted that this latter form is usually characterized by significantly lower intelligence quotients (IQs) [8]. Moreover, classic IQ measures, as obtained by the Wechsler Adult Intelligence Scale-Revised (WAIS-R) [24,25] may potentially vary with the examinee’s physical abilities. As the degenerative and progressive muscles affection is an important feature of muscular dystrophies, one may argue that lower WAIS-R scores could partly relate to muscular weakness, muscular atrophy or myotonia [25].

The objectives of the present study are thus to describe and compare the intellectual abilities of a large sample of DM1 patients with mild and classic adult-onset phenotypes, to estimate the validity of the WAIS-R for the evaluation of intelligence in DM1 patients with muscular weakness, and to appraise the relationship of IQ to CTG repeat length, age at onset of symptoms, and disease duration. Since the striking increase of individuals with a mild phenotype of DM1 in relationship with the availability of genetic counselling and predictive testing [5], it is relevant to better characterize these milder patients as they will represent a very common phenotype of DM1 population in a near future.

## Methods

### Participants

The cohort design included 188 DM1 patients (72 men and 116 women, age range 20–80 years) selected from a study population of 416 mild and classic adult-onset DM1 patients listed at the Saguenay Neuromuscular Clinic registry (Quebec, Canada). Any patient presenting the congenital or childhood onset form of DM1 was excluded, as well as individual with other neurological diseases. Inclusion criteria were age 18 years old or older and being able to give a written informed consent. Study participants were examined by a physiotherapist and had their muscular impairment categorized according to the Muscular Impairment Rating Scale (MIRS) [26]: grade 1, no muscular impairment (*n* = 10, 5.3%): grade 2, minimal signs (*n* = 31, 16.5%); grade 3, distal weakness (*n* = 36, 19.1%); grade 4, mild to moderate proximal weakness (*n* = 91, 48.4%); grade 5, severe proximal weakness (*n* = 20, 10.6%). A patient was considered as having the mild phenotype of DM1 when presenting at least two of the following three criteria: 1- Less than 200 CTG repeats; 2- MIRS grade 1 or 2; 3- Age at onset over 40 years old. To make sure of the accuracy of the information, age at onset of symptoms was noted only if it was precisely and unequivocally given by the patient (*n* = 135). For each participant, the diagnosis was confirmed by a molecular analysis. Mild and classic adult-onset phenotypes were respectively present in 37 (19.7%) and 151 (80.3%) participants. The frequency of the mild phenotype in this patient sample is representative of the entire baseline population at the time of the study [5]. All participants agreed to be visited at home by a neuropsychologist who administered and scored all tests. The evaluation took place in two separate half days in order to minimize mental fatigue. The schedule of testing was standardized across the two days and each participant received the same sequence of testing in order to follow a standardized protocol. Further details regarding this sample as well as the design and survey instruments used are published elsewhere [15,27,28]. This study was approved by the Institutional Review Board of the Centre de santé et de services sociaux de Chicoutimi (Quebec, Canada).

### Neuropsychological measures

#### Wechsler adult intelligence scale-revised (WAIS-R)

Intellectual functioning was evaluated using the French adaptation for the seven-subtest short form of the WAIS-R, given the fact that French is the first language of all participants [24,29,30]. The test is composed of the Information, Digit Span, Arithmetic, and Similarities subtests of the Verbal scale, and of the Picture Completion, Block Design, and Digit Symbol subtests of the Performance scale. The ordinary, non age-corrected scaled scores are included into algorithms to prorate the weighted sums of Verbal and Performance scaled scores [29]. United States (US) adult population-based norms were used. The mean ± SD is 100 ± 15 for the IQ indices, and 10 ± 3 for the subtests scaled scores [24]. The composite reliability estimates of VIQ, PIQ, and FSIQ for the seven-subtest short form are respectively .96, .94, and .97 compared to the entire WAIS-R [29].

The administration of every subtests was not possible for 29 patients (11 men, 18 women; aged between 32 and 77 years; 22 classic adult-onset and 7 mild phenotype; (CTG)n ranging from 50 to 1900), mostly due to fatigue reported by the patients themselves and caused by a sustained mental effort throughout the overall evaluation protocol. However, in these cases, the computation of the estimated FSIQ score was still possible by using a three-subtest short form, based on the administration of the Information, Digit Span, and Picture Completion subtests [31]. The composite reliability estimates of FSIQ using these three subtests is excellent (.91) [31], even if this combination does not permit the calculation of a prorated VIQ and PIQ.

#### Raven’s standard progressive matrices (SPM)

To make sure that low WAIS-R FSIQ measures in DM1 patients are unlikely to represent a bias due to muscular weakness of the upper limbs, the SPM [32] were also administered. The SPM is a widely used intelligence test designed to measure nonverbal reasoning ability, and many studies have demonstrated the SPM’s efficacy as a measure of general intelligence by showing strong positive correlations with the Wechsler tests [33]. Predicted WAIS-R FSIQ (FSIQ’) scores were derived from total raw scores on the SPM using a regression equation [34].

### Statistical analyses

Mann–Whitney U Test was used as most of the data did not meet the assumption of normal distribution. One-sample t-test was used to determine whether two distributions of the same sample of DM1 patients were different one from another. The degree of the linear relationship between two variables was assessed by Spearman’s correlation. Finally, chi-square tests were used to compare the frequency of cases between two categorical variables. Statistical significance was assumed at the level of *p* < 0.05. Data were analysed using the IBM SPSS Statistics version 20 for Mac OS.

## Results

### DM1 participants

From the 416 DM1 patients listed at the Saguenay Neuromuscular Clinic registry, a total of 82 (19.7%) were excluded from the study for reasons such as no longer lives in the SLSJ region (36.6%), incorrect contact information (25.6%), refusing clinical follow-up (20.7%), and suffering from another major health problem (17.1%). Another group of 131 (31.5%) potential participants refused to participate. Of these, 77 (58.8%) were not interested, 28 (21.4%) invoked employment, health or time issues, 16 (12.2%) had speech or mobility limitations, and 10 (7.6%) gave other reasons. Of the 203 (48.8%) DM1 patients who consented to take part to the study, 3 dropped out. Comparisons between the 200 DM1 patients who participated in the study and the 216 nonparticipants showed no differences in terms of sex, CTG repeat length, and proportion of mild versus classic adult-onset phenotype of DM1, but the two groups slightly differed in terms of age (47.0 ± 11.8 years vs 50.2 ± 14.6 years respectively, *p* < 0.05). Finally, 12 participants did not underwent the WAIS-R (7 men and 5 women, 5 mild and 7 classic adult-onset phenotype, age range 38–80 years, CTG repeat length range 90–1900, education range 4–14 years).

Table 1 shows clinical characteristics of the cohort study of 188 DM1 patients. As expected, mild phenotype patients were significantly older and had a higher age at onset of symptoms, a lower disease duration and a lower number of CTG repeats than classic adult-onset phenotype patients. Years of education and the proportion of men and women did not differ between groups. Comparisons between patients who completed the seven-subtest short form of the WAIS-R (*n* = 159) and those who did not (*n* = 29), revealed that age, education, age at onset of symptoms, disease duration, (CTG)n and MIRS were not different, as well as for the proportion of men and women, and mild and classic adult-onset phenotypes of DM1. However, the DM1 patients who did not complete the WAIS-R obtained a lower FSIQ (77.4 vs 83.6, *p* < 0.001).

**Table 1:** **Clinical profile of patients with DM1**

**Characteristics**	**Total**	**Classic adult-onset**	**Mild**	***P*** **value**
	***n*** **=188**	***n*** **=151**	***n*** **=37**	
Age, y	45.7 ± 11.0	43.5 ± 9.0	54.9 ± 13.7	<0.001
Sex M:F	72:116	60:91	12:25	n.s.
Education, y	9.9 ± 2.6	9.8 ± 2.5	10.3 ± 2.7	n.s.
Age at onset, y^a^	21.6 ± 9.4	20.4 ± 7.6	43.7 ± 12.8	<0.001
Disease duration, y^a^	22.4 ± 8.9	23.0 ± 8.5	10.0 ± 5.4	<0.001
(CTG)n	811.3 ± 524.5	973.3 ± 449.2	149.8 ± 168.0	<0.001
MIRS	3.4 ± 1.1	3.7 ± 0.8	2.3 ± 1.1	<0.001

Mean ± SD.

DM1 = Myotonic Dystrophy type 1; (CTG)n = Cytosine-Thymine-Guanine repeats size;

MIRS = Muscular Impairment Rating Scale.

^a^Data available for 135 DM1 patients; 128 classic adult-onset and 7 mild form.

### Wechsler adult intelligence scale-revised

Tables 2 and 3 present WAIS-R IQs and the mean scaled scores of the seven WAIS-R subtests of DM1 patients with mild and classic adult-onset forms. WAIS-R FSIQ scores ranged from 61 to 119, with a median score of 82. For both mild and classic adult-onset phenotypes, all three mean IQ indices are below the general population mean (except for PIQ in the mild group). Despite their low mean IQs, the mild form group had a higher mean FSIQ, a higher mean VIQ, and a higher mean PIQ compared to the classic adult-onset form group. Irrespectively of the DM1 form, all subtests mean scaled scores are below the normative sample mean. On the other hand, results indicate that mild phenotype patients had a higher performance than classic adult-onset phenotype patients on Digit Span, Arithmetic and Block Design subtests (Table 3).

**Table 2:** **WAIS-R IQs of patients with DM1**

**WAIS-R IQs**	**Total**	**Classic adult-onset**	**Mild**	***P*** **value**
	***n*** **=188 (159** ^**a**^ **)**	***n*** **=151 (129** ^**a**^ **)**	***n*** **=37 (30** ^**a**^ **)**	
FSIQ	82.6 ± 8.4	81.1 ± 7.5	88.7 ± 9.2	<0.001
VIQ	83.4 ± 9.1	82.3 ± 9.0	87.8 ± 8.5	0.001
PIQ	85.7 ± 10.2	83.6 ± 8.2	94.8 ± 12.8	<0.001
VIQ-PIQ discrepancies	7.9 ± 6.3	7.2 ± 6.0	10.8 ± 6.7	0.002

Mean ± SD.

WAIS-R = Wechsler Adult Intelligence Scale-Revised; IQ = Intelligence Quotient;

DM1 = Myotonic Dystrophy type 1; FSIQ = Full-Scale IQ; VIQ = Verbal IQ; PIQ = Performance IQ.

^a^Valid N for VIQ and PIQ indices.

**Table 3:** **Comparisons of mean scaled scores for the seven-subtest WAIS-R in classic adult-onset and mild DM1 patients**

**Scale**	**Subtest**	**Total**	**Classic adult-onset**	**Mild**	***P*** **value**
Verbal	Information	6.5 ± 1.8 (187)	6.3 ± 1.8 (151)	7.0 ± 2.1 (36)	n.s.
	Digit Span	6.0 ± 2.4 (188)	5.7 ± 2.4 (151)	6.8 ± 2.3 (37)	0.045
	Arithmetic	7.5 ± 2.4 (179)	7.2 ± 2.3 (144)	8.6 ± 2.3 (35)	0.004
	Similarities	7.0 ± 2.2 (168)	6.9 ± 2.2 (135)	7.1 ± 2.1 (33)	n.s.
Performance	Picture Completion	6.7 ± 2.2 (188)	6.6 ± 2.1 (151)	7.1 ± 2.5 (37)	n.s.
	Block Design	6.3 ± 2.2 (186)	6.0 ± 2.0 (150)	7.5 ± 2.6 (36)	<0.001
	Digit Symbol	6.6 ± 2.1 (187)	6.5 ± 2.0 (150)	6.9 ± 2.5 (37)	n.s.

Mean Scaled Scores ± SD (*n*).

WAIS-R = Wechsler Adult Intelligence Scales-Revised; DM1 = Myotonic Dystrophy type 1.

Table 2 also shows the absolute VIQ-PIQ mean discrepancies, ranging from 0 to 29, and from 0 to 31 for mild and classic adult-onset DM1 patients, respectively. The former showed a greater variability in their PIQ scores (72 to 124) as compared with the classic adult-onset DM1 patients (65 to 111). Conversely, the mild group showed a lesser variability in their VIQ scores (68 to 112) than classic adult-onset DM1 patients (58 to 109). All this could explain the higher VIQ-PIQ mean discrepancies in favour of the mild DM1 patients. Altogether, results suggest that the mean PIQ of DM1 patients is significantly higher (*p* < 0.01) than the mean VIQ. Moreover, the VIQ-PIQ discrepancies were in favour of a PIQ > VIQ pattern for more than 60% of the total sample (*n* = 159). Finally, about 15% of all DM1 patients showed a difference of 15 points (one SD) or more between their VIQ and PIQ scores.

**Figure 1:**
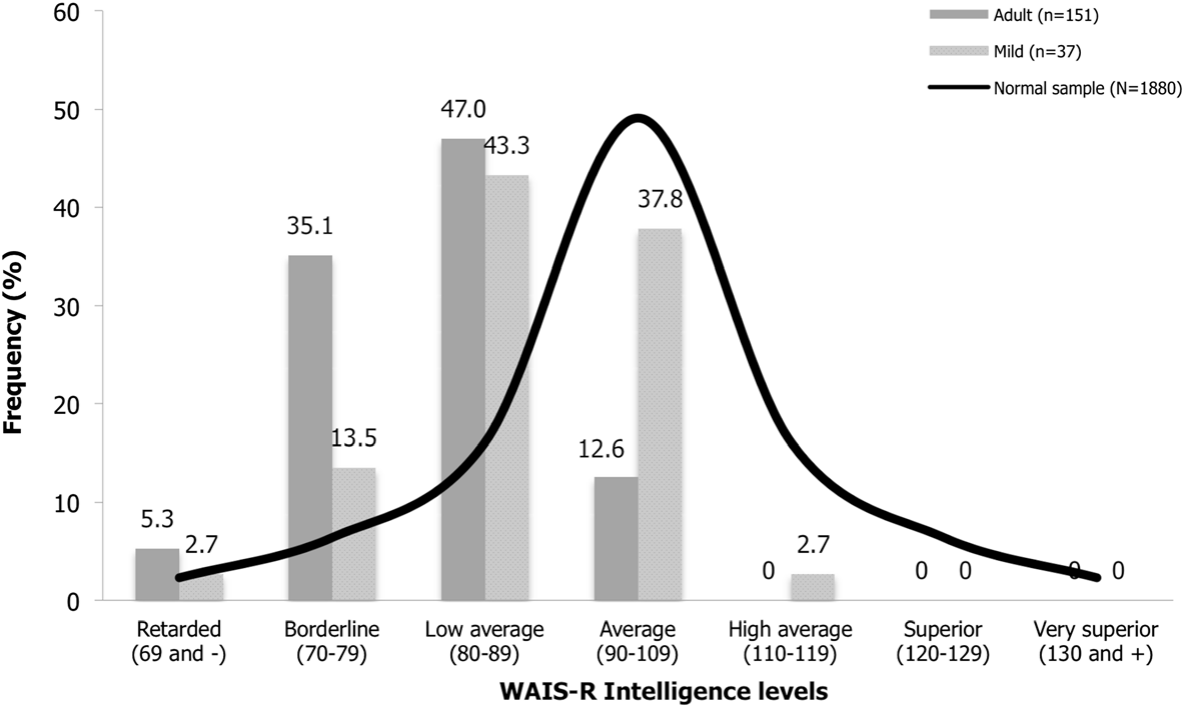
**Distribution of intelligence levels based on WAIS-R FSIQ in classic adult-onset and mild DM1 patients compared to normal sample.**

Figure 1 illustrates the distribution of intelligence levels based on WAIS-R FSIQ scores for mild and classic adult-onset DM1 patients. This figure shows a clear asymmetry in both FSIQ distributions towards a left-sided displacement compared to normal sample. More particularly, classification of FSIQ scores showed that 87.4% of the classic adult-onset and 59.4% of the mild DM1 patients were below the average range (90–109) of intellectual functioning. Figure 1 further shows that the proportion of classic adult-onset DM1 patients scoring in the WAIS-R retarded range of intellectual functioning is more than twice the proportion of the normal population.

**Figure 2:**
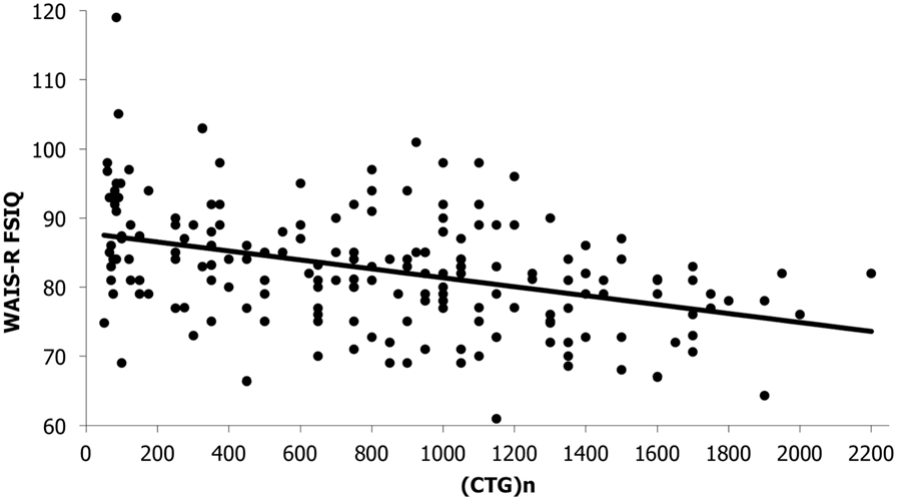
**Correlation analysis between WAIS-R FSIQ and CTG repeats size in patients with DM1.**

**Figure 3:**
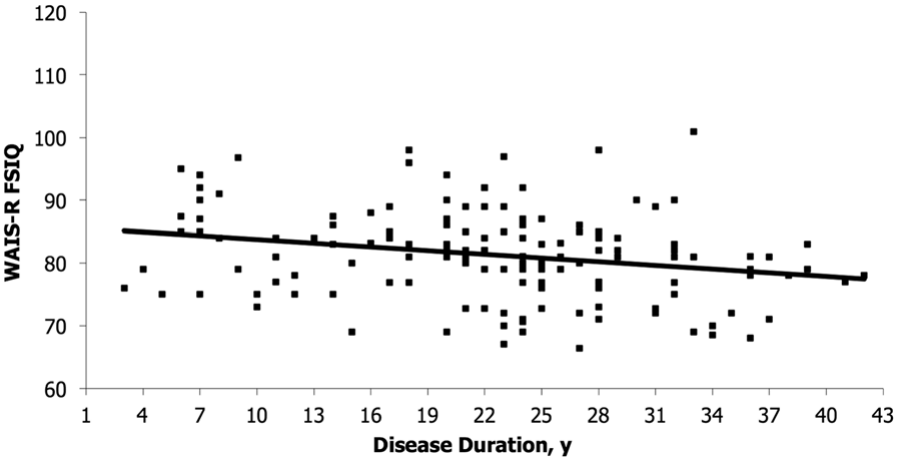
**Correlation analysis between WAIS-R FSIQ and disease duration in patients with DM1.**

Figures 2 and 3 present the relationship between WAIS-R FSIQ and CTG repeats size and disease duration for the total sample. There is a statistical significant decline in FSIQ scores with both the increase of the (CTG)n (*r*_*s*_ = −0.41, *p* < 0.001) and the disease duration (*r*_*s*_ = −0.26, *p* = 0.003), even if they are considered moderate and weak correlation, respectively [35]. Finally, FSIQ was not linked to the age at onset of symptoms.

### Standard progressive matrices

Table 4 compares the distributions of the WAIS-R diagnostic labels according to FSIQ measurement and SPM FSIQ’ estimations. The overall mean ± SD for the WAIS-R FSIQ and the SPM estimation are 82.6 ± 8.4 and 81.4 ± 7.8, respectively. Except for one case (Average 90-109), there are no significant differences between mean WAIS-R FSIQ and mean SPM estimation within each diagnostic labels. Moreover, there is a strong significant correlation between these two measures of intelligence for the whole sample (*r*_*s*_ = 0.67, *p* < 0.001), and within each subgroup of DM1 patients (mild: *r*_*s*_ = 0.43, *p* = 0.007; classic adult-onset: *r*_*s*_ = 0.70, *p* < 0.001).

**Table 4:** **Distributions of WAIS-R diagnostic labels according to WAIS-R FSIQ measurement and SPM FSIQ’ estimation in DM1 patients**

	**WAIS-R FSIQ**			**SPM FSIQ’**			
	**(** ***n*** **=188)**			**(** ***n*** **=187)**			
**WAIS-R diagnostic labels**	**Frequency**	**Valid percent**	**Mean ± SD**	**Frequency**	**Valid percent**	**Mean ± SD**	***p*** **Value** ^**a**^
Very superior (130 and above)	_	_	_	_	_	_	_
Superior (120–129)	_	_	_	_	_	_	_
High average (110–119)	1	0.5	119.0	_	_	_	_
Average (90–109)	33	17.6	94.6 ± 3.7	31	16.6	93.1 ± 3.0	0.008
Low average (80–89)	87	46.3	84.0 ± 2.7	72	38.5	84.5 ± 3.3	n.s.
Borderline (70–79)	58	30.9	75.3 ± 3.0	74	39.6	75.5 ± 2.8	n.s.
Deficiency (69 and below)	9	4.8	67.0 ± 2.8	10	5.3	66.9 ± 2.7	n.s.

WAIS-R = Wechsler Adult Intelligence Scales-Revised; FSIQ = Full-Scale IQ; SPM = Standard Progressive Matrices.

^a^Differences between mean WAIS-R FSIQ and mean SPM FSIQ’ for each WAIS-R diagnostic labels were calculated using a one-sample t-test where the known value was the corresponding mean WAIS-R FSIQ.

## Discussion

This study evaluated the intellectual abilities of a large sample of DM1 patients with mild and classic adult-onset forms. Reduced intelligence associated with DM1 has been mentioned in many studies including the first descriptions of the disease [36]. Intellectual impairment of different degrees has been described in previous reports but some of them can be criticized for as being biased relatively to the inclusion of congenital DM1 patients or small sample sizes [8–10,12,37]. The present study offers more reliable estimates of intellectual functioning as we used a large and uniform cohort of DM1 patients with mild and classic adult-onset phenotypes, with the exclusion of any congenital or childhood onset patients. Our results first demonstrate that intelligence level of mild and classic adult-onset DM1 patients is characterized by a low general intellectual functioning in both verbal and nonverbal abilities. For our total sample (*n* = 188), the mean WAIS-R FSIQ was 82.6, corresponding to low average IQ according to Wechsler [24]. By comparison, a study including 17 classic adult-onset DM1 patients gave similar results on a full WAIS administration, with a FSIQ of 85.3 (VIQ = 87.2; PIQ = 84.1) [19]. Moreover, a recent report [21] on a sample of 121 DM1 patients revealed a mean IQ of 85.0 on an abbreviated WAIS-III, which is also similar to our results. It should nonetheless be mentioned that this latter study included 7.9% of juvenile phenotype. Our findings also contrasted with other previous studies of classic DM1 patients reporting a mean total FSIQ of 94.2 (*n* = 37) [12], 95.0 (*n* = 47) [38] or 96.7 (*n* = 23) [39], which corresponds to average intelligence levels. In addition, one study reported a median FSIQ of 91 on 14 classic adult-onset DM1 patients [40], while another observed a mean FSIQ of 98.8 from a SPM calculation on 50 DM1 patients [41]. Our study does not validate these findings. Regarding the FSIQ, standard deviations ranged from +1.27 to −2.60 SD, and 64.4% (*n* = 121) had a FSIQ significantly lower (>1 SD) than the normative sample mean. Moreover, almost 5% of the patients obtained an IQ in the intellectual disability range, which is more than twice the percent of the WAIS-R standardization sample. Only one patient had an IQ in the high average range of intellectual functioning.

Studies that measured IQ in mild DM1 patients have generally not detected any defect in global intelligence. For example, a group of 36 late-onset DM1 patients showed a mean SPM IQ of 107.4 [11], while 13 mild form patients obtained a mean IQ of 98.9 (range 83–116) on a two-subtest abbreviated WAIS-R [13]. In the present study, 59.4% of our mild DM1 patients (*n* = 37) were below the average range of intellectual functioning, namely low average (*n* = 16, 43.3%), borderline (*n* = 5, 13.5%), and intellectual disability (*n* = 1, 2.7%). One could argue that these divergent findings regarding mild phenotype could be explained by differences in levels of fatigue or excessive daytime sleepiness (EDS). To clarify this issue, one of our previous study on the same cohort of patients provide interesting data concerning clinical correlates associated with presence or absence of fatigue and/or EDS [15]. All DM1 participants were categorized into four distinct groups according to their excessive fatigue and EDS level. Post hoc comparisons showed that FSIQ varies neither with excessive fatigue nor EDS status. Another partial explanation may come from the group of 29 patients who did not complete all subtests of the WAIS-R short form. Their FSIQ scores were based on a lower composite reliability estimates than the one of the seven-subtest short form (.91 vs .97 compared to the entire WAIS-R), which led to a significant lower FSIQ. Despite this previous assumption, we believe that this could have a negligible impact on the results, considering the presence of only 7 mild DM1 patients in that group, and an equal proportion of mild and classic adult-onset phenotypes in both groups.

The definition of a mild phenotype of DM1 also varies from one study to another. The original terminology by Koch et al. [42] classified as “mild” patients those with an age at onset over 40 years old. The definition used in the present study took advantage of the deoxyribonucleic acid (DNA) analysis results and is a combination of a small expansion of CTG repeats, the absence of muscular impairment or a late age at onset. From a clinical perspective, the combined use of neuromuscular and genetic criteria allow a more sensitive characterization of these milder patients. Using a definition of mild cases based only on a small expansion of less than 200 CTG repeats, we also observed many patients with FSIQ scores below the average range of intellectual functioning as shown in Figure 2. In all, our results strongly suggest that intellectual impairment is an extremely common and important feature in DM1, not only among the classic adult-onset patients but also among the least severe forms of DM1, with low IQ scores compared to general reference population.

Our DM1 patients also obtained significant low scores on every WAIS-R subtests, with better scores on Digit Span, Arithmetic, and Block Design for the mild group. Moreover, we found a significant relationship between FSIQ and (CTG)n. This can partly explain the fact that the mild group of DM1 patients obtained higher IQ indices. In fact, patients with more CTG repeats have a lower IQ than those with smaller expansions (*r*_*s*_ = −0.41, *p* < 0.001). This is consistent with previous studies who noted an association between higher CTG repeat size and some cognitive impairment in classic adult-onset DM1 patients [19,21,38]. Such significant negative relationships with FSIQ have also been described in severe and mild congenital DM1 children [43] and childhood [43,44] forms of DM1. In combination with these reports, our study on classic adult-onset and mild DM1 portrays the natural course of this complex multisystemic neuromuscular disorder throughout a clinical continuum in its CNS manifestations. We believe that these results are further evidence of an existing overlap between the different phenotypes in DM1. From this point of view, higher IQs and better scores on some subtests in favour of the mild group of patients could rather reflect a later onset of the disease than a complete distinct cognitive phenotype. We also found that FSIQ was not linked to the age at onset of symptoms. For patients affected by DM1, the symptoms reported at onset are usually myotonia, distal weakness or both [45]. In the present study, the absence of such correlation suggests that there is no close relationship between the muscular involvement and the CNS impairment as measured by the FSIQ. This suggestion of two separate processes in DM1 evolution has also been recently described in a study that analysed cerebrospinal fluid levels of three biomarkers in patients with DM1, namely total tau (T-tau), phosphorylated tau (P-tau) and 42-amino-acid form of *ß*-amyloid (A*ß*_42_*)*. Although the A*ß*_42_/P-tau ratio was decreased in classic adult-onset DM1 patients, along with a tendency of increased T-tau and P-tau levels, the authors did not find any correlation between these biomarkers and disease duration or severity of muscular involvement [46]. DM1 is a progressive disease affecting the muscles, but the question if CNS dysfunction in DM1 is also a progressive condition remains by cons uncertain. It was suggested that progressive CNS dysfunction could potentially account for cognitive decline rather than a true neurodegenerative process per se [47]. The present results showed that FSIQ scores significantly decreased with the increase of the disease duration. This finding may support the hypothesis of a certain global decline in cognitive abilities in DM1 over time but needs to be confirmed by longitudinal designs. A follow-up study (mean follow-up: 7.3 ± 2.7 years) on 20 classic adult-onset DM1 patients showed a specific and progressive impairment in attentional capacities, without extension to additional areas of cognition [22]. Over a four-year period, another group of 34 DM1 patients (including 2 congenital forms and 32 classical forms with juvenile or classic adult-onset) showed a significant deterioration in linguistic functions, with a tendency towards decline in executive abilities, confirming a predominant involvement of cognitive functions subserved by fronto-temporal areas of the brain [23].

It is well acknowledged that educational attainment is highly correlated to psychometric measures of intelligence [24]. In this context, as IQs between mild and classic adult-onset DM1 patients are quite similar, it is not surprising to observe that years of education are the same for both groups. One of our previous study also demonstrated a relationship between increased (CTG)n and higher risk of material and social deprivation [27]. Thus, classic adult-onset DM1 patients are more likely to show higher unemployment, lower family income, and higher reliance on social assistance compared with mild DM1 patients. One could argue that this discrepancy of the social impact between mild and classic adult-onset DM1 patients is probably explained by the lower motor skills of the latter. Since classic adult-onset DM1 patients obtained the lowest IQs, with a high frequency in the intellectual disability range, it is reasonable to believe that intellectual impairment is an important contributing factor in the socioeconomic deprivation portrayed in patients with DM1 [27]. From our clinical experience, additional factors such as daytime sleepiness, fatigue, personality traits, apathy or other cognitive deficits could also contribute to the vulnerability of these patients in experiencing socioeconomic inequalities.

Intellectual evaluation, as for any neuropsychological investigation, depends in some way on motor abilities, which may be impaired in DM1. We used SPM as a motor-independent test of intelligence to assess muscular impairment as a possible confounding factor in the establishment of WAIS-R IQ indices. We found a strong correlation between total WAIS-R and SPM FSIQ estimation, both for the entire sample and within each subgroup of DM1 patients. Moreover, our results showed a discrepancy in favour of a significant higher mean PIQ score compared to VIQ, added to a more frequent PIQ > VIQ pattern. These results altogether suggest that the WAIS-R is a valid instrument to evaluate global intelligence in mild and classic adult-onset DM1 patients, on the one hand, and that muscular weakness in DM1, particularly of the upper limbs, relates not to intellectual impairment, on the other. Moreover, our results are in agreement with a previous study showing that both verbal and nonverbal WAIS subtests were consistently lower in a group of classic adult-onset DM1 patients compared to normal and neurological controls such as spinal muscle atrophy, suggesting that cognitive impairment is not a consequence of motor impairment [19].

This study has some limitations that should be addressed in future researches. First, assessment of adaptive skills was not included. In addition to better characterize our DM1 patients with very poor IQ scores, a complete assessment of the adaptive behavior would have been useful in determining how well these individuals respond to daily demands from the environment. Also, our study does not include unaffected family members of the households of the patient or case controls. Despite that our results are compared to population-based norms, the inclusion of a family member unaffected by DM1 could have clarified if low IQ scores are linked to the disease or if they are a consequence of any socioeconomic deprivation previously reported in DM1 population [27]. In our study, IQ scores were obtained using the French adaptation of the WAIS-R [30], although this version is not validated in a French Canadian population. A French European standardization sample exists but we decided to use US norms since we consider that these data are more likely to represent the intellectual levels of North-American adults. A study who compared the French and American WAIS-R standardization samples showed that the two are very similar, except for two variables. Education level is slightly lower for the French subjects, while the French sample included an age group (75–79 years) that is missing from the American sample, which leads to give a greater weight to the variable Age [48]. Finally, whenever possible, future studies in DM1 could correlate their neuropsychological data to neuroimaging or functional investigations such as PET (positron emission tomography) or SPECT (single-photon emission computed tomography).

## Conclusions

The impact of DM1 on brain and cognition is well described. It is generally acknowledged that intelligence is lower in this population as compared to normal control subjects, and that intellectual disability is characteristic of the congenital and childhood onset forms of the disease. Our findings showed that intellectual impairment is an extremely common and important feature of DM1, and could also be observed in individuals mildly affected by DM1, with low IQ scores compared to the general reference population. Since the introduction of predictive DNA testing, the proportion of patients with a mild phenotype is an increasing phenomenon in DM1 [5], and health care providers involved in the follow-up of these patients should be aware of their intellectual capacities and should adapt their interventions accordingly.

### Study approval

This study was approved by the Institutional Review Board of the Centre de santé et de services sociaux de Chicoutimi.

### Patient consent

All participants gave their written informed consent prior to their inclusion in the study.

## Abbreviations

A*ß*42: 42-amino-acid form of *ß*-amyloid
CNS: Central Nervous System
CTG: Cytosine-Thymine-Guanine
DM1: Myotonic Dystrophy type 1
DNA: Deoxyribonucleic acid
FSIQ: Full-Scale IQ
IQ: Intelligence quotient
MIRS: Muscular impairment rating scale
P-tau: Phosphorylated tau
PIQ: Performance IQ
SD: Standard deviation
SLSJ: Saguenay-Lac-Saint-Jean
SPM: Standard progressive matrices
T-tau: Total tau
US: United States
VIQ: Verbal IQ
WAIS-R: Wechsler adult intelligence scale-revised
